# The effects of traditional Chinese medicine combined with chemotherapy on immune function and quality of life in patients with non-small cell lung cancer: A protocol for systematic review and meta-analysis

**DOI:** 10.1097/MD.0000000000022859

**Published:** 2020-11-06

**Authors:** Li-Na Zhao, Yin-Qing Yang, Wen-Wen Wang, Qian Li, Hua Xiao

**Affiliations:** a School of Health Preservation and Rehabilitation; b School of Management, Chengdu University of Traditional Chinese Medicine, Sichuan, China.

**Keywords:** chemotherapy, immune function, non-small cell lung cancer, quality of life, randomized controlled trials, traditional Chinese medicine

## Abstract

**Background:**

This article will evaluate the effects of traditional Chinese medicine (TCM) combined with chemotherapy on the immune function and quality of life of patients with non-small cell lung cancer (NSCLC), and evaluate the published side effects.

**Methods:**

The systematic review and meta-analysis will be conducted in accordance with the Preferred Reporting Items for Systematic Review and Meta-Analysis guidelines. The databases we will search include: PubMed, EMBASE, Cochrane Library, Web of Science, China National Knowledge Infrastructure, China Biomedicine, Wan fang Data, and Technology Periodical Database. The search date is from inception to June 30, 2020. There are no restrictions on the document language. The literatures included in this study are randomized controlled trials. The main results include ratio of CD3^+^, CD4^+^, CD8^+^, CD4^+^/CD8^+^, NK cells, the level of IgA, IgG, IgM, and Karnofsky performance status score. The secondary result is to evaluate various side effects during treatment. We will use the Cochrane Collaboration tool to evaluate each study and use Review Manager software (RevMan, version 5.3) to merge and analyze the data. The 2 researchers will independently cross-screen the literature, extract data, and evaluate the quality. If there are differences, we will resolve them through discussion or consultation with a third reviewer.

**Results:**

The results of this study will provide high-quality evidence for the effect of TCM combined with chemotherapy on the immune function and quality of life of patients with NSCLC.

**Conclusion:**

This article will comprehensively evaluate the effects of TCM combined with chemotherapy on the immune function and quality of life of patients with NSCLC, and provide evidence-based evidence for clinical practice.

**Ethics:**

Since the data used in this study is based on previous trials and does not involve patient privacy, ethical approval is not required.

**Study registration number:**

INPLASY202070071

## 1 Introduction

Lung cancer is one of the most common malignant tumors in humans, and its morbidity and mortality are among the highest in the world. According to statistics, about a quarter of cancer deaths are due to lung cancer, and its 5-year survival rate is only 19%.^[1,2]^ The study have showed that in 2018, lung cancer in China accounted for 21.9% of male malignant tumors and 13.3% of female malignancies,^[3]^ which posed a serious threat to human health, and at the same time brought a heavy burden to families and society. Among them, non-small cell lung cancer (NSCLC) accounts for about 80% to 85%of all lung cancers,^[4,5]^ and because of its occult onset, it is difficult to detect in the early stage. Most patients have been diagnosed as advanced,^[6]^ and missed the best opportunity for surgery. Therefore, chemotherapy has become one of the main treatment methods for advanced NSCLC.^[7,8]^ However, most of the chemotherapeutics are cytotoxic periodic drugs with weak selectivity, which not only kill tumor cells, but also damage normal cells to varying degrees, resulting in further impairment of patients’ immune function, and their toxic side effects also greatly affect patients’ quality of life.^[9–11]^ Therefore, it is essential to find drugs that can alleviate the suppression of immune function by chemotherapy.

Modern studies have found that the occurrence of lung cancer is closely related to the abnormal immune status,^[12]^ and the body's low immunity will weaken the body's anti-cancer ability and promote the development of lung cancer, which is similar to what traditional Chinese medicine (TCM) calls “where pathogenic qi gathers, healthy qi must be deficient”.^[13]^ TCM emphasizes strengthening the body and eliminating pathogens in the treatment of lung cancer, and exerts anti-tumor effects by regulating autoimmune function. A large number of clinical trials have shown that TCM can inhibit the development of lung cancer through a variety of ways, with unique advantages such as improving the body's immunity, prolonging the survival of patients, improving clinical symptoms and improving the quality of life of patients.^[14–16]^ The study found that the combination of TCM and chemotherapy can not only enhance the anti-cancer effect, but also reduce the adverse reactions after chemotherapy, which can significantly enhance the immune function and improve the quality of life of lung cancer patients.^[17,18]^ However, there is no relevant systematic review to study the effect of TCM combined with chemotherapy on the immune function and quality of life of patients with NSCLC. Therefore, this article aims to provide evidence-based evidence for the effect of TCM combined with chemotherapy on the immune function and quality of life of patients with NSCLC.

## 2 Method

**Study registration**

This study has been registered on the INPLASY platform with number INPLASY202070071. We will report this study based on the Preferred Reporting Items for Systematic Review and Meta-Analysis Protocols.^[19]^

### 2.1 Inclusion criteria

#### 2.1.1 Types of studies

1) The literatures included in this study are all randomized controlled trials, and there is no language restriction;
2) we can extract valid data or find full text;
3) the article deals with the effect of Chinese medicine combined with chemotherapy on immune function and quality of life of NSCLC.

#### 2.1.2 Types of participants

Patients with NSCLC diagnosed pathologically and age≥18 years old will be included, and there are no restrictions on gender and race.

#### 2.1.3 Types of interventions

We will include all patients who receive TCM combined with chemotherapy in the treatment of NSCLC.

#### 2.1.4 Type of comparators

The intervention in the control groups is chemotherapy alone.

### 2.2 Types of outcome measures

#### 2.2.1 Primary outcomes

1) Evaluation of immune function: a) ratio of CD3^+^, CD4^+^, CD8^+^, CD4^+^/CD8^+^, NK cells. b) levels of IgA, IgG and IgM.
2) Quality of life assessment: Karnofsky score (Karnofsky performance status) will be used as the criterion to evaluate the changes of patients’ quality of life.

#### 2.2.2 Secondary outcomes

Evaluation of toxic and side effects: according to the WHO anti-tumor drug toxic reaction criteria, it is divided into 0 to 4 grades to evaluate various toxic and side effects during treatment.

### 2.3 Exclusion criteria

1) Duplicate articles or data;
2) The full text cannot be obtained through various channels;
3) Animal research or subjects;
4) Participants who had non-pathological diagnosis, previously received chemotherapy, radiotherapy or surgery, or other malignant tumors;
5) Other TCM methods such as acupuncture, massage, and cupping.

## 3 Search methods for the identification of studies

The databases we will search include: PubMed, EMBASE, Cochrane Library, Web of Science, China National Knowledge Infrastructure, China Biomedicine, Wan fang Data, and Technology Periodical Database. Search terms included “non-small cell lung cancer”, “traditional Chinese medicine”, “chemotherapy”, “immune function”, “quality of life”, “randomized controlled trial” and so on. We will search the references of published reviews and relevant conference records to identify potential eligible studies. The search date is from inception to June 30, 2020. There are no restrictions on the document language and publication status. Table 1 is an example of a PubMed database search strategy.

**Table 1:** Search strategy sample used in PubMed.

Number	Search terms
1	Carcinoma, Non-Small-Cell Lung
2	Carcinoma, Non Small Cell Lung
3	Carcinomas, Non-Small-Cell Lung
4	Lung Carcinoma, Non-Small-Cell
5	Lung Carcinomas, Non-Small-Cell
6	Non-Small-Cell Lung Carcinomas
7	Nonsmall Cell Lung Cancer
8	Non-Small-Cell Lung Carcinoma
9	Non Small Cell Lung Carcinoma
10	Carcinoma, Non-Small Cell Lung
11	Non-Small Cell Lung Cancer
12	1 OR 2 OR 3 OR 4 OR 5 OR 6 OR 7 OR 8 OR 9 OR 10 OR 11
13	Traditional Chinese medicine
14	Chinese medicine
15	Chinese herbal medicine
16	Proprietary Chinese medicine
17	13 OR 14 OR 15 OR 16
18	chemotherapy
19	chemotherapies
20	chemotherapeutic
21	18 OR 19 OR 20
22	Randomized controlled trial
23	controlled clinical trial
24	randomized
25	controlled
26	trial
27	random
28	placebo
29	22 OR 23 OR 24 OR 25 OR 26 OR 27 OR 28
30	12 AND 17 AND 21 AND 29

## 4 Data collection and analysis

### 4.1 Selection of studies

**Figure 1:**
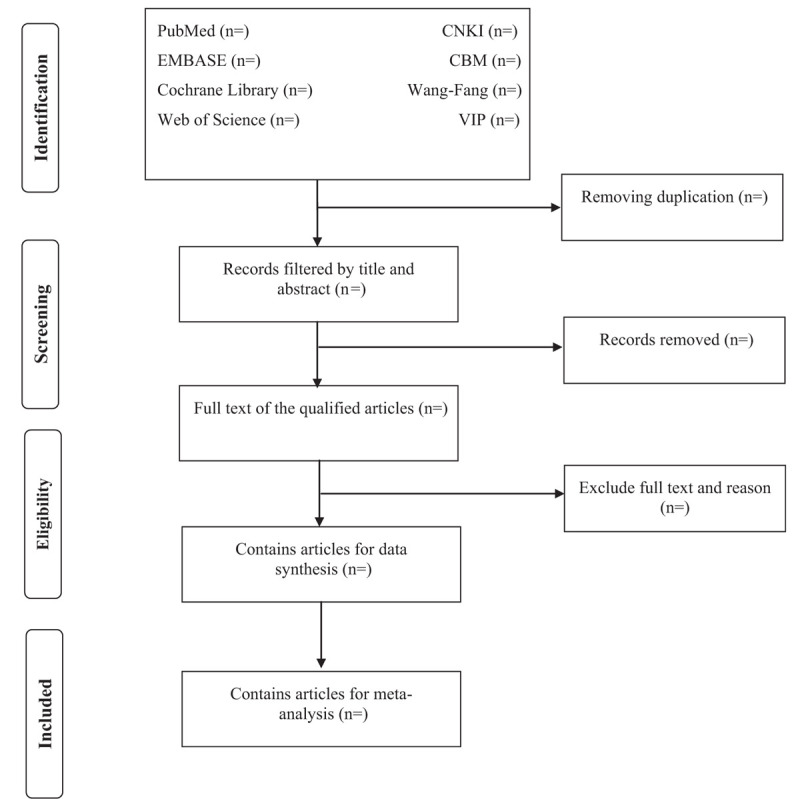
Flow diagram of studies selection.

We will use Endnote X9 (Thomson Scientific, Connecticut, USA) to manage the literature. After eliminating the repetitive literature, 2 reviewers (LNZ and QL) independently screened the title and abstract, and then read the full text to determine the studies that met the criteria. Any disagreements regarding the choice of research will be discussed and resolved by the 2 reviewers and, if necessary, by the third reviewer (WWW). All details of the research selection will be shown in the following PRISMA diagram (Fig. 1).

### 4.2 Data extraction

All included studies will be independently extracted by 2 researchers (LNZ and YQY) according to the pre-designed form. The extracted content includes: first author, publication year, nationality, research period, patient age, gender, sample size, tumor node metastasis stage, Chinese medicine intervention, chemotherapy regimen, treatment time, outcome indicators, etc. In case of missing data, we will contact the authors for accurate data. If there is disagreement between the 2 researchers, we will resolve it through group discussion and, if necessary, by a third researcher (QL).

### 4.3 Assessment of risk of bias in included studies

The risk of bias will be assessed independently by 2 reviewers (QL and WWW) using the Cochrane Collaboration tool,^[20]^ which includes the following 7 items: random sequence generation, allocation concealment, blinding of participants, and personnel, blinding of outcome assessment, incomplete outcome data, selective outcome reporting, and other bias. The bias risk of each project is divided into “low”, “unclear”, and “high risk of bias”. If 2 researchers have different opinions, they will be resolved through discussion or consultation with the third reviewer (LNZ).

### 4.4 Data synthesis

We will use Review Manager software (RevMan, version 5.3) to merge and analyze the data. Relative risk will be used to analyze the results for binary data. For continuous data, mean difference or standard mean deviation will be used to analyze the results. The confidence interval for both binary and continuous variables is set to 95%. The heterogeneity of the included studies will be assessed by the I^2^ statistic. If there is no obvious heterogeneity, the fixed effects model will be used for analysis; if I^2^≥50%, it indicates that the heterogeneity is large, and we will use the random effects model. At the same time, sensitivity analysis and subgroup analysis will be conducted to explore the source of heterogeneity. When meta-analysis is not available, the results will be reported in a narrative manner.

#### 4.4.1 Subgroup analysis

We will conduct a subgroup analysis of age, gender, treatment cycle, and tumor node metastasis stage.

#### 4.4.2 Sensitivity analysis

To ensure the reliability and stability of the results, we will conduct a sensitivity analysis to assess the impact of studies with high risk of bias.

#### 4.4.3 Assessment of reporting bias

If the number of included trials is sufficient, we will use the funnel chart to evaluate report deviations and small-study effects. The more obvious the asymmetry of the funnel chart, the greater the possibility of publication bias.^[21,22]^

#### 4.4.4 Dealing with missing data

If any information is missing or unclear, we will contact the corresponding author via email. If the author cannot be contacted, we will analyze the currently available data and discuss the potential impact of missing data.

#### 4.4.5 Assessment of heterogeneity

The heterogeneity will be evaluated by I^2^ statistics.^[23]^ There is no significant heterogeneity when *P* > .1 and I^2^ < 50%, and fixed effect model will be used for data synthesis. When *P* ≤ .1 and I^2^ ≥ 50%, it indicates that there is significant heterogeneity, and random effect model will be used for data synthesis. Analyze the source of heterogeneity through subgroup analysis and sensitivity analysis.

#### 4.4.6 Grading the quality of evidence

Grading of Recommendations Assessment, Development, and the Evaluation scoring system will be used to assess the quality of evidence for the main results.^[24]^ The quality of evidence is divided into 4 levels: high, medium, low, and very low.

### 4.5 Ethics and dissemination

This study does not involve the privacy of patients and does not require ethical approval. The research results will be published in a peer-reviewed journal.

## 5 Discussion

The incidence of lung cancer is closely related to the contrast of the body's positive and pathogenic forces. Deficiency of healthy qi and low immune function are the key factors for recurrence and metastasis of lung cancer. TCM has a long history in the treatment of lung cancer, focusing on the whole, strengthening the healthy qi and eliminating pathogens, superficial and radical. That is to say “keep healthy, do not be evil”.^[25]^ In the comprehensive treatment of lung cancer, TCM exerts anti-cancer effects by improving the immune function of the body and other ways,^[26]^ thereby prolonging the survival period of patients and improving the quality of life. The combination of TCM and chemotherapy can not only enhance the ability to kill tumor cells, but also reduce the recurrence and metastasis of lung cancer, alleviate the gastrointestinal tract and other adverse reactions after chemotherapy, and can largely alleviate the pain of patients and improve the quality of life, which is widely used in clinical practice. However, as far as we know, there is no systematic review related to the effects of TCM combined with chemotherapy on the immune function and quality of life of patients with NSCLC. Therefore, we conducted a systematic review of the effects of TCM combined with chemotherapy on the immune function and quality of life of patients with NSCLC. The results of this study may be beneficial for clinicians to treat NSCLC.

## 6 Limitations

In this study, only Chinese and English databases will be searched, so there may be limitations, leading to omission of relevant information.

## Author contributions

**Conceptualization:** Lina Zhao, Hua Xiao.

**Data curation:** Lina Zhao, Yinqing Yang, Qian Li, Wenwen Wang.

**Methodology:** Hua Xiao, Lina Zhao, Yinqing Yang, Qian Li.

**Resources:** Lina Zhao, Qian Li, Wenwen Wang.

**Supervision:** Lina Zhao, Yinqing Yang, Hua Xiao.

**Writing – original draft:** Lina Zhao.

**Writing – review & editing:** Lina Zhao, Yinqing Yang, Hua Xiao.

All authors read and approved the final manuscript. Hua Xiao is the guarantor of this review.
